# Design and Synthesis of TiO_2_ Hollow Spheres with Spatially Separated Dual Cocatalysts for Efficient Photocatalytic Hydrogen Production

**DOI:** 10.3390/nano7020024

**Published:** 2017-01-25

**Authors:** Qianqian Jiang, Li Li, Jinhong Bi, Shijing Liang, Minghua Liu

**Affiliations:** 1 Department of Environmental Science and Engineering, Fuzhou University, Minhou 350108, China; jqq199207@163.com (Q.J.); lili@fjpsc.edu.cn (L.L.); mhliu2000@fzu.edu.cn (M.L.); 2 State Key Laboratory of Photocatalysis on Energy and Environment, Fuzhou University, Fuzhou 350002, China

**Keywords:** TiO_2_ hollow spheres, dual cocatalysts, spatial separation, hydrogen production

## Abstract

TiO_2_ hollow spheres modified with spatially separated Ag species and RuO_2_ cocatalysts have been prepared via an alkoxide hydrolysis–precipitation method and a facile impregnation method. High-resolution transmission electron microscopy studies indicate that Ag species and RuO_2_ co-located on the inner and outer surface of TiO_2_ hollow spheres, respectively. The resultant catalysts show significantly enhanced activity in photocatalytic hydrogen production under simulated sunlight attributed to spatially separated Ag species and RuO_2_ cocatalysts on TiO_2_ hollow spheres, which results in the efficient separation and transportation of photogenerated charge carriers.

## 1. Introduction

Semiconductor photocatalysis as a green technology has attracted much interest for the application in solving environmental pollution and energy shortage [1,2,3,4,5]. Since the photolysis of water to produce hydrogen was discovered, TiO_2_ has been most investigated in photocatalysis due to the chemical stability, nontoxicity, and low price [5,6,7,8]. However, the drawbacks of TiO_2_, such as the low utilization of sunlight, the rapid recombination of the photogenerated charges, and few suitable active sites, extremely limit photocatalytic performance. Tuning the morphology and structure of TiO_2_ with expectations of achieving novel or enhanced properties have been regarded as an efficient way to overcome the drawbacks—for instance, the fabrication of TiO_2_ nanospheres, nanorods, nanowires, and nanobelts [9,10,11,12,13]. Especially, the submicron-scale hollow spheres of TiO_2_ are promising because they can provide large specific surface areas and enhance light scattering properties and their inner and outer surfaces can be controlled and selectively functionalized [14]. Moreover, the photocatalytic properties of TiO_2_ hollow spheres can be modified by loading cocatalysts [15,16,17], which can serve as reaction sites and provide the trapping sites for the photogenerated carriers of the surface. The internal electric field can be formed between the cocatalyst and the photocatalyst due to the different Fermi level, which can promote the directional migration of photogenerated electrons and holes and prohibit the recombination of the photogenerated carriers [18].

The space locations of the cocatalysts loaded on the photocatalytic materials can greatly affect the migration of the photogenerated carriers and then further affect the photocatalytic activity. The oxidation cocatalyst and reduction cocatalyst loaded on different spatial locations of the photocatalysts may produce spatially separated reaction sites with oxidizing and reducing abilities, respectively, which consequently lead to the directional migration of photogenerated electrons and holes and thus prohibit the recombination of photogenerated carriers. Domen et al. have demonstrated that SiO_2_/Ta_3_N_5_ core/shell structures with spatially separated cocatalysts show superior photocatalytic activity [19]. Li et al. have reported that reduction cocatalysts (MoS_2_, NiS, WS_2_, etc.) and oxidation cocatalysts (IrO*_x_*, MnO*_x_*, RuO*_x_*, etc.) can be selectively deposited on the (010) and (110) facets of BiVO_4_, respectively, which results in much higher photocatalytic activity compared to that with randomly distributed cocatalysts [20,21]. In general, the noble metals (Au, Ag, Pt, Pd, etc.), MoS_2_, and graphene exhibiting superior electron mobility often act as reduction cocatalysts to improve the efficiency of photoproduction electron migration [7,22,23,24,25]. The cocatalysts such as RuO_2_, IrO_2_, CoO*_x_*, and MnO*_x_* can act as hole collector [26,27,28,29]. Loading the reduction and oxidation catalysts on the inner and outer surfaces of TiO_2_ hollow spheres can be expected to achieve enhanced photocatalytic activity.

**Scheme 1:**
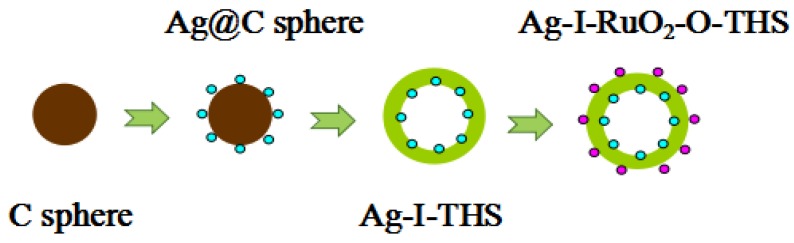
Processes involved in the formation of dual cocatalysts co-loading on the TiO_2_ hollow spheres (THS).

Herein, we report a facile synthesis of TiO_2_ hollow spheres modified with spatially separated Ag species and RuO_2_ on the inner and outer surfaces of the TiO_2_ hollow spheres (as shown in Scheme 1), which exhibited enhanced photocatalytic hydrogen production under solar light irradiation.

## 2. Experimental Section

### 2.1. Preparation of Catalysts

#### 2.1.1. Synthesis of Carbon Spheres (C Sphere)

In a typical synthesis of carbon spheres, glucose (6 g) was dissolved into deionized water (60 mL) to form a clear solution and then was transferred into a 100 mL Teflon-lined autoclave and was reacted at 180 °C for 12 h. The obtained brown product was collected and washed with deionized water and ethanol and then dried at 80 °C. Finally, carbon spheres (denoted as C sphere) were obtained [30].

#### 2.1.2. Synthesis of TiO_2_ Hollow Spheres (THS)

An amount of 0.4 g of C sphere was added to 30 mL of pure ethyl alcohol. The obtained suspended solution was stirred for 30 min and then was dispersed under ultrasonic conditions for 30 min. Then, a solution consisting of 70 mL of pure ethyl alcohol, 0.2 g of hexadecyl trimethyl ammonium bromide (CTAB), and 0.6 mL of deionized water was added and stirred for 2 h. After that, 23 mL of a tetrabutyl titanate ethanol solution was added dropwise while stirring. The obtained suspended solution was transferred into the three-necked flask. After the reflux condensation at 85 °C for 100 min, the prepared product was collected, washed with deionized water and ethyl alcohol, and dried at 60 °C. The dried powders were further calcined at 500 °C for 2 h with a ramping rate of 2 °C/min and TiO_2_ hollow spheres were then obtained and are denoted as THS [30].

#### 2.1.3. Synthesis of Ag-Loaded TiO_2_ Hollow Spheres on the Inner Surface (Ag-I-THS)

The loading Ag on the inner surface of THS included two steps. In the first step, Ag-loaded C sphere was prepared by an impregnation method [31]. An amount of 0.4 g of the above synthesized carbon spheres were impregnated in a 0.4 mL silver nitrate solution (10 mg/mL) and then dried at 80 °C for 2 h. The resulting powders were reduced by excess NaBH_4_ solution (0.1 M). The obtained product was washed with deionized water and ethanol and dried at 60 °C. Finally, the Ag-loaded carbon sphere powders (denoted as Ag@C sphere) were obtained. The second step was similar to the synthesis of THS except that 0.4 g of C sphere was replaced by 0.4 g of Ag@C sphere in this process. After the treatment, the Ag-loaded TiO_2_ hollow spheres on the inner surface were obtained and are denoted as Ag-I-THS.

#### 2.1.4. Synthesis of Ag- and RuO_2_-Co-Loaded TiO_2_ Hollow Spheres on the Inner Surface and Outer Surface (Ag-I-RuO_2_-O-THS)

The above synthesized Ag-I-THS (0.2 g) was impregnated in a 0.2 mL ruthenium chloride solution (10 mg/mL) and then dried at 80 °C for 2 h. The resulting powders were calcined at 350 °C for 1 h, and Ag- and RuO_2_-co-loaded TiO_2_ hollow spheres on the inner surface and outer surface, respectively, were finally obtained and are denoted as Ag-I-RuO_2_-O-THS.

#### 2.1.5. Synthesis of RuO_2_-Loaded TiO_2_ Hollow Spheres on the Outer Surface (RuO_2_-O-THS)

The loading of RuO_2_ on the outer surface of THS was also conducted by an impregnation process [31]. The above synthesized THS (0.4 g) was impregnated in a 0.4 mL ruthenium chloride solution (10 mg/mL) and then dried at 80 °C for 2 h. The resulting powders were calcined at 350 °C for 1 h, and RuO_2_-loaded TiO_2_ hollow spheres on the outer surface were finally obtained and are denoted as RuO_2_-O-THS [32].

#### 2.1.6. Synthesis of RuO_2_- and Ag-Co-Loaded TiO_2_ Hollow Spheres on the Inner Surface and Outer Surface (RuO_2_-I-Ag-O-THS)

The loading of RuO_2_ and Ag on the inner surface and outer surface of THS included three steps. In the first step, RuO_2_-loaded carbon spheres were prepared by an impregnation method [31]. An amount of 0.4 g of the above synthesized carbon spheres were impregnated in a 0.4 mL ruthenium chloride solution (10 mg/mL) and then dried at 80 °C for 2 h. The resulting powders were calcined at 350 °C for 1 h, and the RuO_2_-loaded carbon sphere powders (denoted as RuO_2_@C sphere) were finally obtained. The second step was similar to the synthesis of THS, except that the 0.4 g of carbon spheres were replaced by 0.4 g of RuO_2_@C sphere in this process. After the treatment, the RuO_2_-loaded TiO_2_ hollow spheres on the inner surface were obtained and are denoted as RuO_2_-I-THS. In the final step, the above synthesized RuO_2_-I-THS (0.2 g) was impregnated in a 0.2 mL silver nitrate solution (10 mg/mL) and then dried at 80 °C for 2 h. The resulting powders were reduced by excess NaBH_4_ solution (0.1 M). The obtained product was washed with deionized water and ethanol and dried at 60 °C. Finally, RuO_2_- and Ag-co-loaded TiO_2_ hollow spheres on the inner surface and outer surface, respectively, were obtained and are denoted as RuO_2_-I-Ag-O-THS.

### 2.2. Characterizations

The as-prepared samples were characterized by powder X-ray diffraction (PXRD) on a Rigaku Mini Flex 600 X-ray diffractometer (Rigaku, Akishima, Japan) operated at 40 kV and 15 mA with Ni-filtered Cu Kα irradiation (λ = 1.5406 Å). Solid-state UV-Vis diffuse reflectance spectra (UV-Vis DRS) were obtained by using a UV-Vis spectrophotometer (Varian, Cary 500, Palo Alto, CA, USA). Barium sulfate was used as a reference. The Brunauer–Emmett–Teller (BET) surface area was measured with an ASAP2020 apparatus (Micromeritics, Atlanta, GA, USA). The transmission electron microscopy (TEM) images were recorded using a JEOL model JEM 2010 EX microscope (FEI, Hillsboro, OR, USA) at an accelerating voltage of 200 kV. X-ray photoelectron spectroscopy (XPS) measurements were performed on a PHI Quantum 2000 XPS system (Physical electronics, Portland, OR, USA) with a monochromatic Al KR source and a charge neutralizer. All binding energies were referenced to the C 1s peak (284.6 eV) of the surface adventitious carbon.

### 2.3. Photocatalytic Activity Evaluation

Photocatalytic hydrogen evolution from water-splitting reaction was carried out with powder samples to provide sufficient surface area in a glass-closed gas-circulation system and a 100 mL Pyrex glass reaction vessel. The reaction was performed by dispersing 80 mg of catalysts into an aqueous solution (80 mL) containing EDTA-2Na (0.5 g) as a sacrificial electron donor. The whole reaction system was evacuated to completely remove air before irradiation. During the experiment, a 300 W Xe lamp was employed as the light source to simulate sunlight. The incident photon flux is 3.4 × 10^18^ s^−1^·cm^−2^, and the irradiance intensity is 132.4 mW·cm^−2^. The temperature of the reactant solution was kept at a constant temperature by a flow of cooling water during the reaction. The amount of H_2_ evolution was analyzed by using an on-line gas chromatograph (Shimadzu, GC-8A, Kyoto, Japan) with a thermal conductivity detector (TCD) and using argon as the carrier gas. To evaluate the stability of the photocatalyst, the photocatalytic reactions were carried out as the similar procedure above by using 80 mg of catalysts for a total of 25 h with evacuation every 5 h.

## 3. Results and Discussion

### 3.1. Crystal Structure

**Figure 1:**
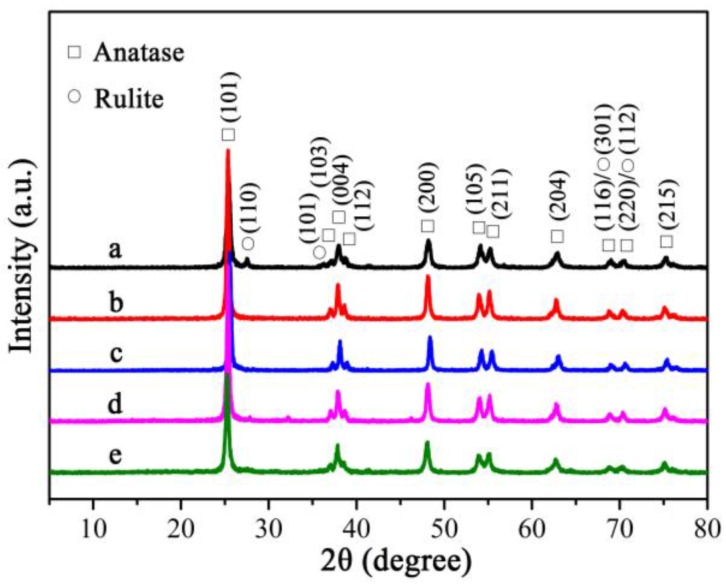
X-ray diffraction (XRD) patterns of TiO_2_ hollow spheres (THS) loaded by the cocatalysts with different spatial dispersion: (**a**) THS; (**b**) Ag-loaded THS on the inner surface (Ag-I-THS); (**c**) RuO_2_-loaded THS on the outer surface (RuO_2_-O-THS); (**d**) Ag- and RuO_2_-co-loaded THS on the inner surface and outer surface (Ag-I-RuO_2_-O-THS); (**e**) RuO_2_- and Ag-co-loaded THS on the inner surface and outer surface (RuO_2_-I-Ag-O-THS).

The XRD patterns of TiO_2_ hollow spheres showed a mixture of anatase and rutile TiO_2_ (Figure 1). The diffraction peak located at 25.3° was attributed to (101) plane of anatase phase, while the diffraction peak located at 27.4° and 36.1° was attributed to (110) and (101) planes of rutile phase [33]. No significant peaks indicative of silver and ruthenium oxide were observed in the cocatalyst-loaded THS, which could be attributed to the very low content and/or high dispersion.

### 3.2. BET Analyses

**Figure 2:**
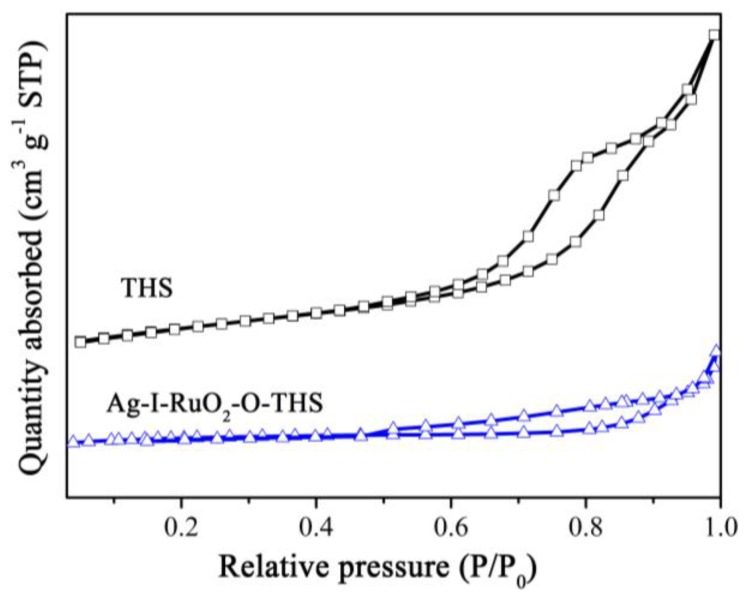
N_2_ adsorption-desorption isotherms of THS and Ag-I-RuO_2_-O-THS.

The BET surface areas and pore structures of THS and Ag-I-RuO_2_-O-THS were evaluated by N_2_ adsorption at 77 K. The pure THS and sample Ag-I-RuO_2_-O-THS displayed type IV N_2_ adsorption–desorption isotherms, corresponding to the mesoporous structure (Figure 2). The BET surface area of THS and Ag-I-RuO_2_-O-THS samples were 34.5 m^2^/g and 8.7 m^2^/g, respectively. Compared with that of THS, the specific surface area of Ag-I-RuO_2_-O-THS was obviously decreased, which may be because the loading of Ag and RuO_2_ blocked off the pores of THS [34].

### 3.3. TEM Analyses

**Figure 3:**
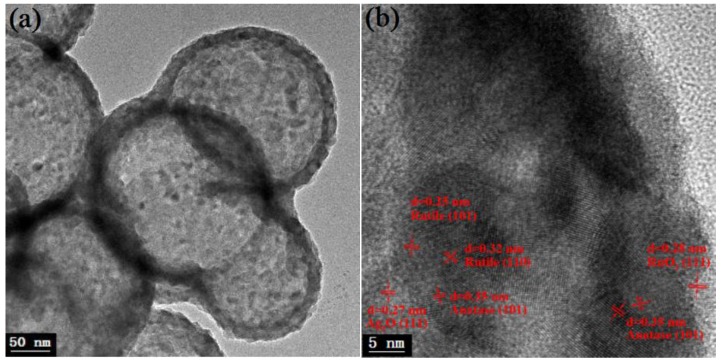
(**a**) Transmission electron microscopy (TEM) and (**b**) High resolution transmission electron microscopy (HRTEM) images of Ag-I-RuO_2_-O-THS.

The morphology of Ag-I-RuO_2_-O-THS and the different spatial distribution of the cocatalysts were investigated by TEM. TEM image of Ag-I-RuO_2_-O-THS clearly elucidated the hollow structure by the striking contrast between the center and the edge with a diameter of ca. 200 nm and a shell thickness of ca. 20 nm (Figure 3a). The HRTEM image (Figure 3b) showed clear lattice fringes. The fringes of *d* = 0.25 nm and 0.32 nm matched well with that of the (101) and (110) crystallographic plane of rutile TiO_2_ and the fringes located at 0.35 nm corresponded to that of (101) crystallographic plane of anatase TiO_2_, which were in accordance with the result of XRD. Noteworthy, the (111) crystallographic plane of Ag_2_O (*d* = 0.27 nm) and the (111) crystallographic plane of RuO_2_ (*d* = 0.28 nm) can be observed on the inner surface and outer surface of TiO_2_ hollow spheres, respectively, indicating the spatially separated dual cocatalysts loaded TiO_2_ hollow spheres has been synthesized successfully.

### 3.4. XPS Analyses

**Figure 4:**
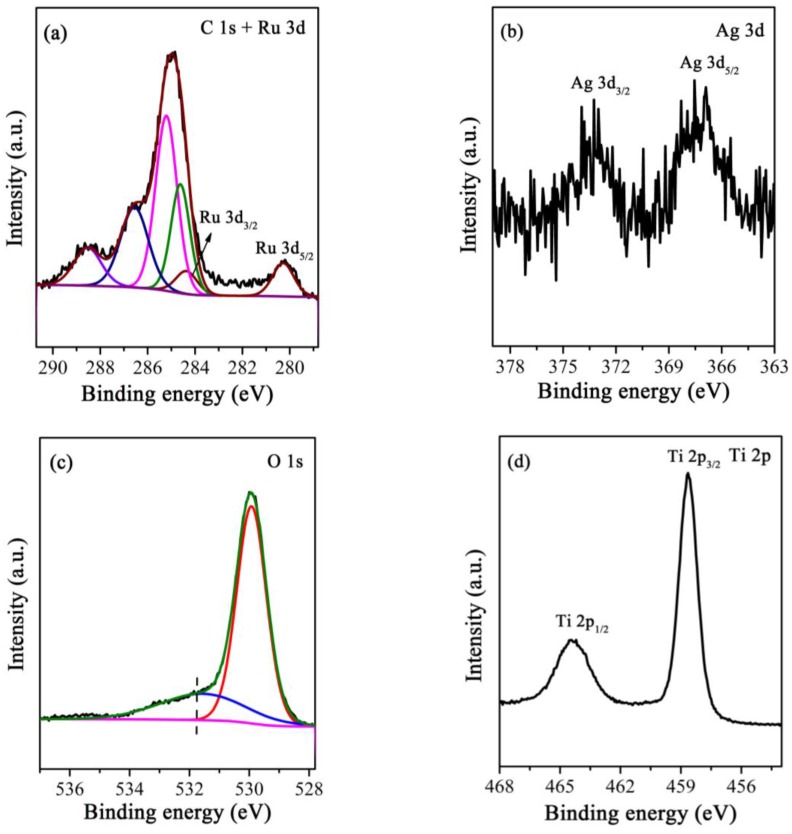
X-ray photoelectron spectroscopy (XPS) spectra of Ag-I-RuO_2_-O-THS: (**a**) C 1s and Ru 3d; (**b**) Ag 3d; (**c**) O 1s; (**d**) Ti 2p.

The chemical components and the states of C, Ru, Ag, O, and Ti in the Ag-I-RuO_2_-O-THS were investigated by XPS. As observed in Figure 4a, there are four peaks at about 284.6 eV, 285.2 eV, 286.6 eV, and 288.5 eV in the C 1s spectrum corresponding to the additional carbon, the residual carbon in C sphere, tetrabutyl titanate, and CTAB, respectively. Furthermore, the binding energy of Ru 3d_3/2_ was overlapped by that of C 1s; thus, the Ru oxidation state was evaluated from the Ru 3d_5/2_. The Ru 3d_5/2_ peak was located at 280.3 eV, which indicated the existence of Ru^4+^, as expected for RuO_2_ [35]. Figure 4b demonstrated the high-resolution XPS spectra for Ag 3d_3/2_ and Ag 3d_5/2_ located at 373.3 eV and 367.3 eV, respectively, corresponding to Ag^+^ of Ag_2_O [36]. The O 1s peak may be fitted into two peaks at 529.9 eV and 531.6 eV (Figure 4c), corresponding to the crystal lattice oxygen and the surface hydroxyl groups, respectively [37]. Meanwhile, the Ti 2p XPS spectra are deconvoluted into two peaks at 458.6 eV and 464.3 eV, corresponding to Ti^4+^ in TiO_2_ [38] (Figure 4d).

### 3.5. UV-Vis DRS Analyses

**Figure 5:**
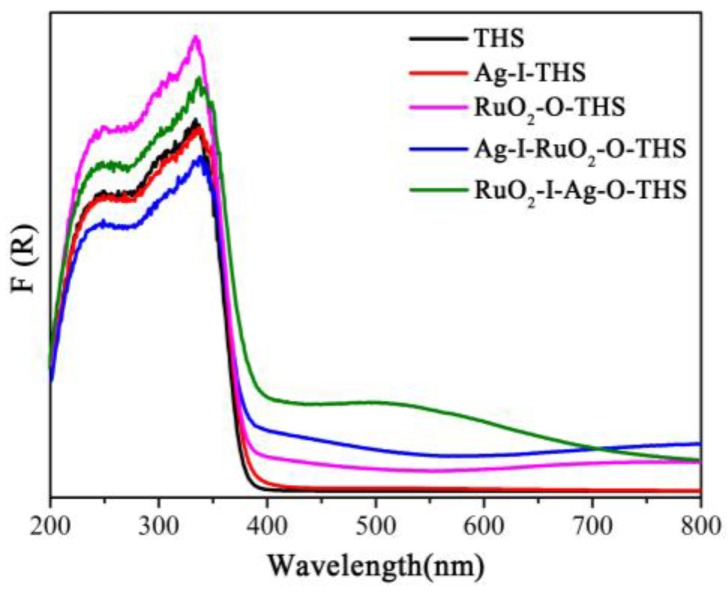
Ultraviolet-visible diffuse reflectance spectra (UV-Vis DRS) of THS loaded by the cocatalysts with different spatial dispersion.

As shown in the UV-Vis diffuse reflectance spectra, all samples displayed a similar band edge with a value of 3.3 eV, indicating that the photo-absorption properties of TiO_2_ were maintained (Figure 5). TiO_2_ absorbed only UV light, while the RuO_2_-O-THS, Ag-I-RuO_2_-O-THS, and RuO_2_-I-Ag-O-THS exhibited a stronger light absorption in the visible light region owing to the presence of RuO_2_. Compared to the other samples, RuO_2_-I-Ag-O-THS composite displayed the characteristic localized surface plasmon resonance peak of Ag located at ca. 530 nm [39].

### 3.6. Photocatalytic Activities

**Figure 6:**
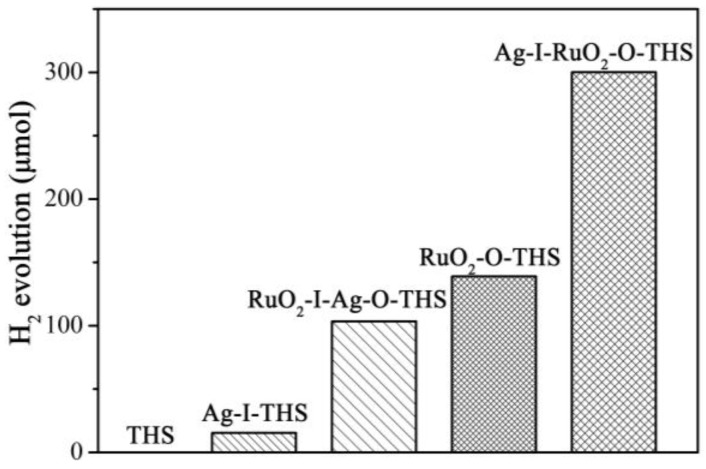
Photocatalytic activities of H_2_ evolution on THS loaded by the cocatalysts with different spatial dispersion.

**Figure 7:**
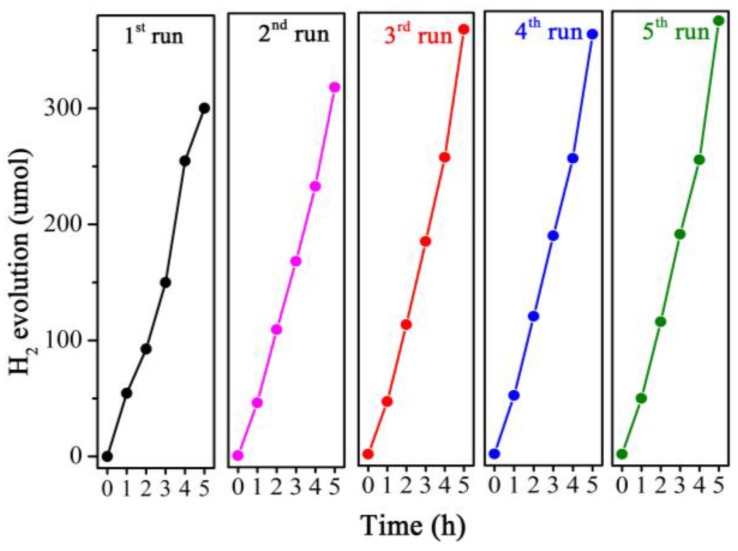
Stability of Ag-I-RuO_2_-O-THS during prolonged photocatalytic operation.

**Scheme 2:**
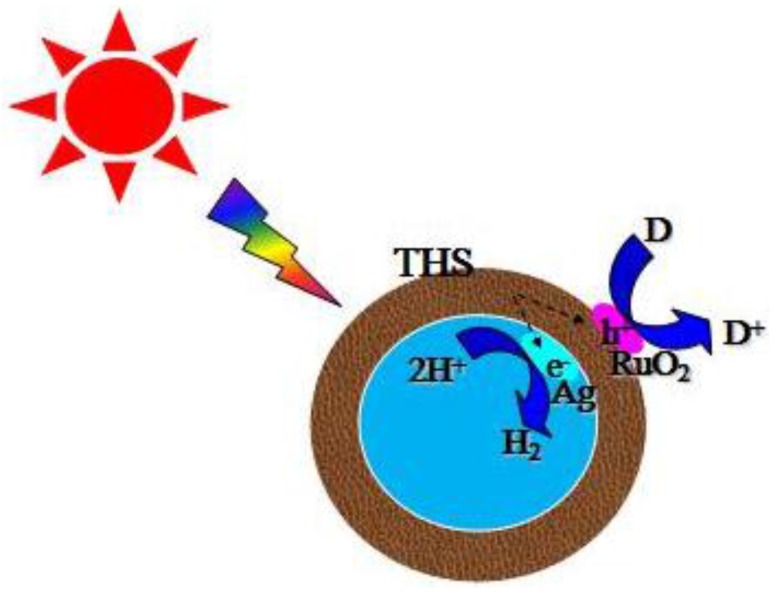
The photocatalytic hydrogen production on Ag-I-RuO_2_-O-THS.

Figure 6 showed the photocatalytic activity of H_2_ evolution of THS loaded with different spatial distribution of the cocatalysts within 5 h. The original TiO_2_ hollow spheres produced a little amount of H_2_. When Ag species and RuO_2_ were loaded on TiO_2_ hollow spheres, the activity of photocatalytic hydrogen production was greatly improved. The hydrogen yields of Ag-I-THS, RuO_2_-O-THS, RuO_2_-I-Ag-O-THS, and Ag-I-RuO_2_-O-THS were 15.4, 139.0, 103.4, and 300.2 μmol, respectively. The H_2_ evolution of Ag-I-RuO_2_-O-THS was about 19.5 times over Ag-I-THS, 2.2 times over RuO_2_-O-THS, and 2.9 times over RuO_2_-I-Ag-O-THS. The results demonstrated that photocatalytic hydrogen production activity was enhanced by co-loading Ag species and RuO_2_ on the inner and outer surfaces of THS. The stability of the photocatalysts is important for their applications. Thus, the stability of Ag-I-RuO_2_-O-THS was investigated. As shown in Figure 7, the photocatalytic activity of Ag-I-RuO_2_-O-THS increased with the increment of the photocatalytic reaction cycles. After three cycles, the hydrogen production tended to be a stable value. The increased amount of hydrogen may be due to the change of the chemical state of silver species under solar light irradiation [36,40]. As the reaction proceeds, the oxidation state of silver species can be gradually reduced to metallic silver, thus increasing photogenerated electrons mobility and significantly improving the hydrogen production. Scheme 2 shows the probable reaction mechanism for the photocatalytic water splitting reaction on Ag-I-RuO_2_-O-THS. The oxidation cocatalyst RuO_2_ and reduction cocatalyst Ag loaded on the outer and inner surface of THS can lead to the directional migration of photogenerated holes and electrons, which can prohibit the recombination of the photogenerated carriers and finally enhance the photocatalytic activity [21].

## 4. Conclusions

We have successfully synthesized TiO_2_ hollow spheres modified with Ag species and RuO_2_ on the inner and outer surfaces, respectively, via an alkoxide hydrolysis-precipitation method combined with a facile impregnation method. The as-obtained TiO_2_ hollow spheres exhibited enhanced photocatalytic hydrogen production activity under solar light irradiation, which is ascribed to the effective transfer and separation of the photogenerated charge carriers at the interface, resulting from the suitable spatial separation of Ag species and RuO_2_ cocatalysts on TiO_2_ hollow spheres.
